# Supplementary material for: Complexome profiling of the Chlamydomonas psb28 mutant reveals TEF5 as an early PSII assembly factor
Source: Plant Cell. 2025 Mar 17;37(6):koaf055. doi: 10.1093/plcell/koaf055 (PMC12164590; doi:10.1093/plcell/koaf055)
Supplement: koaf055_Supplementary_Data [file koaf055_supplementary_data.zip › psb28_interactive.zip!/plots/intensity/qualityPlots/peptides/SNAPA1_Cre02.g099150.t1.2.html]

### Cre02.g099150.t1.2

Trivial name: SNAPA1  
SeqCoverage [median]: 12.90  
Mass: 33.73 kDa  
  
 

| Sequence | MissedCleavage | Mass [Da] | wtIntensity | muIntensity | Score |
| --- | --- | --- | --- | --- | --- |
| AVELFEDAAR | 0 | 1119.56 | 0.00 | 863.23 | 98.03 |
| EIAEQNEK | 0 | 959.46 | 0.00 | 286.22 | 113.89 |
| IAEFSAEMGR | 0 | 1109.52 | 279.05 | 0.00 | 64.71 |
| QAVSIYTDMGR | 0 | 1239.59 | 304.48 | 313.60 | 83.95 |
|  |  |  | 583.5 | 1463.0 |  |
